# Eastward enlargements of the European Union, transitional arrangements and self-employment

**DOI:** 10.1007/s00148-022-00904-2

**Published:** 2022-05-07

**Authors:** Magdalena Ulceluse, Martin Kahanec

**Affiliations:** 1 University of Groningen and GLO, Landleven 1, 9747 AD Groningen, The Netherlands; 2 CELSI, Bratislava, 821 09 Slovakia; 3 Department of Public Policy, Central European University, 1100 Vienna, Austria; 4 GLO, Maastricht, 6211 AX The Netherlands; 5 Faculty of National Economy, University of Economics in Bratislava, Bratislava, 852 35 Slovakia

**Keywords:** Transitional arrangements, EU enlargement, Migration, Self-employment, J10, J15, J18

## Abstract

When the European Union expanded eastward in 2004 and 2007 to accession the so-called EU8 and EU2 countries, respectively, the incumbent member states imposed temporary restrictions on the employment of EU8 and EU2 nationals. Self-employed individuals were exempted from these transitional arrangements, prompting concerns that self-employment could be used as a means to evade the restrictions on labour market access. If the transitional arrangements led to an increase in EU8 and EU2 nationals’ self-employment rates, as previous research suggests, then their removal should have led to a corresponding decrease. This article analyses whether the latter has indeed been the case. Using pooled cross section data from the EU Labour Force Survey, over the period 2004–2019, we show that removing the transitional arrangements has had a negative effect on the self-employment rates of EU2 nationals, but seemingly no effect on the self-employment rates of EU8 nationals. Distinguishing between types of capitalist regimes, however, reveals a much more nuanced picture, with significant variation in terms of the magnitude and significance of the effect across groups of countries.

## Introduction

In 2004 and 2007, the European Union (EU) expanded eastwards to include the countries known as the EU8 and EU2, respectively, granting their citizens the right to work and reside in another member state.^1^ However, citizens of the EU accession countries did not initially receive the same labour market rights as their EU15 counterparts (Drnovšek Zorko and Debnár 2021). Rather, the EU Accession Treaty allowed the incumbent member states to adopt a series of measures that de facto limited access to paid employment for up to seven years for EU2 and EU8 nationals. These measures were called transitional arrangements (TAs) and included a variety of national procedures, such as complex application processes, proof of suitability, work permit requirements, and quotas (Ulceluse and Bender 2022).

Self-employed individuals were not subject to transitional arrangements and could freely access the labour market of any member state. This prompted concerns that self-employment could be used as a means for EU2 and EU8 nationals and their employers to evade the restrictions, with individuals de facto working as employees but registered as self-employed. Moreover, this type of bogus self-employment would be less likely to generate the benefits typically associated with entrepreneurship, i.e., innovation, job creation, and overall contribution to economic growth, less likely to match their level of skills (Ulceluse 2020), and more likely to contribute to the EU2 and EU8 nationals’ labour market precariousness.

If the “self-employment as means of evasion” hypothesis is correct, as existing data (see Section 3) and empirical studies (see Section 2) suggest, at least in certain countries, then we would expect the removal of transitional arrangements to have a negative effect on the self-employment rates of EU2 and EU8 nationals. In other words, if the presence of transitional arrangements contributed to an increase in self-employment for those two migrant groups, their removal should have led to a corresponding decrease.

In this article, we investigate whether this is the case. Specifically, we use pooled cross-section data from the EU Labour Force Survey (EULFS) between 2004 and 2019 to analyse whether and how the removal of transitional arrangements, and thus the granting of full labour market access, had an impact on the self-employment rates of EU2 and EU8 nationals. Our article contributes to the better understanding of the role of labour market policies on migrants’ labour market outcomes and access, and to the literature on the determinants of migrant self-employment.

We structure our article as follows. Section 2 presents an overview of the transitional arrangements implemented following the EU enlargement rounds in 2004 and 2007 and some of their implications. Section 3 presents the data and methodology employed, while Section 4 examines the results. Section 5 discusses the theoretical and policy implications of our findings.

## Transitional arrangements

The transitional arrangements were a series of labour market measures that the EU15 implemented in an effort to manage the potential inflow of workers from the EU8 and EU2 accession countries. The measures themselves were not new, as coordinated restrictions were also implemented when Greece, Spain, and Italy joined the EU. This time, however, the new member states were jointly relatively more populous and differed more significantly in terms of economic development and wages from the incumbent member states. These differences were seen as potentially triggering significant East–West migration. Moreover, some member states were concerned that a migration shock may lead to labour market imbalances by pushing native workers out of their jobs, driving down local wages, and burdening their welfare systems (European Commission 2008; Kahanec and Zimmermann 2010; Kahanec 2013). Consequently, the EU15 member states were allowed, as an exception to the freedom of movement of workers in the European Single Market, to implement temporary measures that would restrict this freedom for workers from the new member states for up to 7 years. The 2 + 3 + 2 rule was agreed at the EU level; however, member states had ample discretion in deciding the length and scope of these restrictions. The rule meant that member states could impose a transitional period for 2 years, then decide whether to extend it for an additional 3 years, and only if there was proof that workers from new member states were causing serious disruptions in the receiving market, the transitional arrangements could be extended for 2 additional years.^2^ The three step rule was established in order to allow for a gradual adjustment of the economic disparities between EU15 countries and the EU8 and EU2 (Holland et al. 2011). Due to a combination of domestic political pressures, economic institutional factors, the positions of other member states (Wright 2010), and the specific socio-economic context and demand for migrant labour, there was significant variation in terms of which countries chose to implement TAs in each enlargement round, and the degree of restrictiveness of those TAs when implemented. We empirically explore this cross-country variation in Section 4 using the varieties of capitalism (Hall and Soskice 2001) framework.

Following the 2004 enlargement, all EU15 member states except for Ireland, Sweden, and the UK implemented TAs for up to 7 years for EU8 nationals. Nevertheless, some member states continued to let in EU8 nationals through bilateral agreements, seasonal work permits, or TA exceptions in particular sectors or occupations, reflecting a largely demand-driven labour migration pattern during this period (Currie 2008). For instance, Austria implemented stringent TAs but issued temporary work permits to EU8 nationals on the basis of labour market–based needs tests and discretionary decisions of the government, while Germany continued to carry out bilateral seasonal labour agreements which were often utilized by Polish nationals (Tamas and Munz 2006).

Following the 2007 enlargement, and except for Finland and Sweden, all EU15 member states implemented TAs with various degrees of stringency and exemptions. Austria, for instance, introduced work permit exemptions for EU2 nationals working in 65 skilled professions (e.g. bricklayer, paver, data engineer) experiencing a shortage of workers (Groenendijk et al. 2012). France introduced a simplified work permit procedure, and an exemption from needing work permits for students (Fihel et al. 2015), while Germany extended opportunities for seasonal work, contract work, and the posting of workers and labour market access to EU2 nationals with a university degree (Bertoli et al. 2013; 2016). Italy introduced work permit exemptions for various sectors such as agriculture, tourism and hotel business, construction, domestic work and personal assistance, mechanical engineering, management, highly skilled work, or seasonal work (Holland et al. 2011). In the Netherlands, a court ruling essentially removed the need for work permits for seasonal jobs (Groenendijk et al. 2012), while in Spain, EU2 nationals were allowed to work if they were contracted prior to arrival (Drew and Sriskandarajah 2007). In the UK, access to the labour market was strictly regulated and conditional on the possession of a worker authorization card issued for specific jobs only, and on compliance with the national immigration policies in place for low- and high- skilled migrants (Currie 2008).

This variability in terms of the presence/absence, scope, and length of TAs in place for each group of nationals suggests that some member states’ labour markets were more open than others, with implications for the “need” for EU2 or EU8 nationals to turn to self-employment as a way to circumvent barriers to employment. Several studies have specifically investigated whether the implementation of transitional arrangements has in fact affected the self-employment rates of EU2 or EU8 nationals (see, for instance, Boeri and Brucker 2005; Kahanec et al. 2010; Barrell et al. 2010; Clark et al. 2017; Wagner and Hassel 2016). For example, in the UK, Roman (2019) finds that EU2 nationals had a much higher probability to be self-employed than EU8 nationals, while Ruhs and Wadsworth (2018) find that the removal of transitional arrangements in 2014 had a significant and negative effect on the EU2 nationals’ propensity to become self-employed. Taken together, these two studies indicate that EU2 nationals (and UK employers) might have indeed used self-employment strategically as an entryway into the UK labour market. In Germany, post enlargement EU8 nationals were likelier to be self-employed than employees (Brenke et al. 2010), and by some estimates up to five times more likely to be self-employed than previous cohorts (Elsner and Zimmermann 2016). Similarly, in Austria, the number of self-employed Polish nationals increased four times, and doubled for the EU8 population as a whole between 2003 and 2005 (Barrell et al. 2010). These studies seem to lend support to the “self-employment as means of evading transitional arrangements” hypothesis for EU8 nationals too, at least in the countries analysed.

## Data and methodology

### Key variables

In this article, we assess whether and how the removal of transitional arrangements has affected the self-employment rates of EU2 and EU8 nationals in the EU15. Our dependent variables are, thus, the self-employment rates of EU2 and EU8 migrants, computed using the EU Labour Force Survey (EULFS) between 2004 and 2019, as the share of self-employed EU8 and EU2 migrants in the total population of employed EU8 and EU2 migrants, respectively. The EULFS allows us to distinguish between different groups of migrants by country of birth or nationality. We use nationality in this case, as the former is not available in the case of Germany. The quantitative differences between the two are very small or non-existent in most countries. Sweden and Finland do not distinguish between EU2 and EU8 migrants, likely because of the small sample size; therefore, we use the combined group for these two countries.^3^ Although ideally we would distinguish between self-employment with and without employees, under the assumption that the latter is likelier to represent the type of bogus self-employment as hypothesized above, this distinction is not possible using our dataset.

The graphs in Appendix Fig. 1 seem to confirm a relation between the evolution of EU2 and EU8 self-employment rates and the presence or absence of transitional arrangements, at least in certain countries. For instance, in Austria, Belgium, and Germany, the self-employment rates of EU8 nationals increase after the enlargement in 2004 and decrease after 2011, with the removal of transitional arrangements. Similarly, the self-employment rates of EU2 nationals decrease in countries such as Austria, Germany, the Netherlands, and the UK after the removal of TAs, post-2014.

Our two main independent variables are meant to capture the effect of the removal of transitional arrangements on self-employment rates. To that end, we define them as two dummy variables equal to 1 for the period after the end of transitional arrangements for the EU8 and EU2 nationals, respectively, by country. Table 1 presents the year when each country removed the TAs for each migrant group.

**Table 1:** Period of transitional arrangements by country for EU8 and EU2 nationals

Country	EU-8	EU-2
Austria	2004–2011	2007–2014
Belgium	2004–2009	2007–2014
Denmark	2004–2009	2007–2009
Finland	2004–2006	*No TAs*
France	2004–2008	2007–2014
Greece	2004–2006	2007–2009
Germany	2004–2011	2007–2014
Ireland	*No TAs*	2007–2014
Italy	2004–2006	2007–2012
Luxembourg	2004–2007	2007–2014
Netherlands	2004–2007	2007–2014
Portugal	2004–2006	2007–2009
Spain	2004–2006	2007–2009; 2011–2014*
Sweden	*No TAs*	*No TAs*
UK	*No TAs*	2007–2014

*Spain lifted restrictions for Romania and Bulgaria in 2009, but reintroduced them for Romania in 2011 using the safeguard clause set out in the Accession Treaty

Additionally, we control for a number of factors which might influence both the choice of destination and the opportunities and constraints on the path to become self-employed. We include in our models the share of EU8 and EU2 migrants in the receiving country’s population, to control for potential network and diaspora effects in attracting migrants towards a particular destination, and for the effects of networks on the likelihood to become self-employed. As the graphs in Appendix Fig. 2 illustrate, the EU enlargements led to significant increases in the share of EU8 and EU2 nationals in the EU15 receiving countries’ populations. The increase was mostly incremental in the case of EU8 nationals, although there were a few notable trends in Denmark, Ireland, the Netherlands, and the UK. In the UK and especially Ireland, both of which did not implement TAs, the share of EU nationals soared post-2004, while in Denmark and the Netherlands, it increased sharply post-2009 and post-2011, respectively, the years in which they removed the transitional arrangements. Similarly, the share of EU2 nationals in the total population of Italy and Spain grew significantly throughout the period analysed, regardless of the presence or the absence/removal of transitional arrangements in place. This development likely reflects the many TA exceptions present in these two countries, particularly in sectors experiencing labour shortages. On the other hand, in countries such as Belgium, Denmark, France, Greece, Luxembourg, or Portugal, which implemented more restrictive measures, the share of EU2 nationals increased sharply only after the removal of the transitional arrangements.

We also include the self-employment rate of the native population, as a proxy for the overall entrepreneurial culture and the friendliness of institutions and regulations to self-employment in the destination country. Furthermore, our models include three other variables which have been strongly linked to self-employment rates: unemployment, GDP per capita, and employment protection legislation. High unemployment may affect self-employment positively as the opportunity cost of starting a business decreases, or negatively, as it also entails fewer resources available which could undermine the creation of new businesses (see for example Blau 1987; Blanchflower and Meyer 1994; Audretsch et al. 2002; and for an extensive review Thurik et al. 2008). We obtain unemployment rates for the entire active population in each EU15 country, between 2004 and 2019, from Eurostat (2021b). The level of GDP per capita in purchasing power adjusted, a proxy for economic development, may affect self-employment negatively if it is associated with greater capital per worker, or if the returns from waged employment relative to self-employment are now higher (Lucas 1978). Conversely, it can have a positive effect on self-employment, when it is the result of increased economic growth, demand for goods and services and access to credit, encouraging business creation (Parker and Robson 2004). We obtain data on GDP per capita from the Eurostat (2021a) for the period 2004–2019. Self-employment rates might also be affected by the stringency of employment protection regulations (see Ulceluse and Kahanec 2018). By virtue of their role, that of protecting employees from dismissal, wage loss, or unfair treatment from employers, labour market regulations might make hiring and firing costlier. This in turn might incentivize employers to contract out work to individuals, therefore increasing self-employment rates. In order to control for this effect, we include in our models a variable reflecting the strictness of employment protection on individual and collective dismissals for regular contracts, obtained from the OECD database on employment protection. The indicators are compiled using the OECD’s own reading of statutory laws, collective bargaining agreements and case law as well as contributions from officials from OECD member countries and advice from country experts (OECD 2020).

Table 2 provides an overview of the main characteristics of the variables employed in our analysis.

**Table 2:** Summary statistics of main variables

Variable	Obs	Mean	Std. dev
EU2 self-employment rate	238	12.470	12.179
EU8 self-employment rate	237	11.499	7.228
Native self-employment rate	239	15.885	7.758
Unemployment	240	8.577	4.699
GDP per capita	240	32,439.58	11,637.39
Employment protection legislation	236	2.334	0.7119

Own computations using the EULFS

Lastly, migrants’ propensity to become self-employed may depend on the national socio-economic context and institutional configuration and the opportunities and constraints they create. In order to account for this potential variation in self-employment rates, and keeping in mind the limited number of observations in our dataset, we turn to the Varieties of Capitalism (VoC) literature (Ulceluse 2016; Bechter et al. 2012; Hall and Soskice 2001), as one of the most influential explanations for variation in economic outcomes across countries. The VoC literature emphasizes how institutions relating to finance, employment, welfare, industrial relations, and education and training evolve differently in each country and how the interaction between them translates into different models of capitalism (Dilli et al. 2018). These models, which are fairly stable over time, can help explain quantitative and qualitative variation in the supply of migrant labour over time and across space, and migrants’ subsequent economic outcomes (Devitt 2011). We thus include in our model a dummy variable that accounts for the four types of capitalist regimes in our sample, namely, Liberal (Ireland, UK), Continental (Austria, Belgium, Germany, Luxembourg, Netherlands), Nordic (Denmark, Finland and Sweden), and Southern (France, Greece, Italy, Portugal and Spain).

### Empirical model

To assess the effect of the removal of TAs on the self-employment rates of EU2 and EU8 nationals, we estimate the following model:

1 $${Y}_{it}={\beta }_{0}+{\beta }_{1}{X}_{ i t}+{\beta }_{1}{Z}_{ i t}+{\varepsilon }_{it}$$

where ${Y}_{it}$ is the dependent variable, either self-employment rates for EU2 or self-employment rates for EU8 migrants, $X$ represents the independent variable capturing the post-transitional arrangements period, ${\beta }_{1}$ is its slope, $t$ refers to the time unit — years, $i$ to the cross-national units — countries, while $\varepsilon$ is the error term. $Z$ represents a vector of the control variables described above.

Using the EU LFS and other sources of data, we constructed a longitudinal dataset of the EU15 countries for the period of 2004–2019 on which we estimate Eq. 1 using the fixed-effects and random-effect panel estimators. In order to decide on the appropriate model for our data, we conducted a series of specification tests. We begin with a Hausman (1978) test, which assesses whether the errors (*ui*) are correlated with the regressors, with the null hypothesis being that they are not. The tests suggest that the random effects estimator is consistent both in case of the EU2 model (*p* = 0.993) and in case of the EU8 model (*p* = 0.303). The Wooldridge test for serial correlation indicates that the residuals are autocorrelated in both the EU2 (*p* = 0.028) and EU8 (*p* = 0.022) models. Lastly, a Breusch-Pagan Lagrange multiplier test for contemporaneous correlation suggests that residuals are correlated across countries in the same cross-section (*p* = 0.000 for both models). Based on these results, we proceed to employ a fixed effects model with robust standard errors clustered at the country level, which corrects for these deviations and allows for a better inference using time series cross-sectional data. In order to control for the influence of aggregate time series trends, we employ time fixed effects across all our models.

## Analysis

Table 3 presents the results of our analysis for the EU2 migrant group. We start with a parsimonious model 1, which only includes our dependent variable and a proxy for the effect of the enlargement, to which we add our control variables in model 2. Both models 1 and 2 seem to confirm our expectations that the removal of transitional arrangements has had a negative effect of the self-employment rates of EU2 nationals within the EU15 incumbent member states.

**Table 3:** The effect of TAs removal on EU2 self-employment rates

Variables	(1)	(2)	(3)
TA EU2	− 4.304*	− 3.627*	− 2.629
	(2.054)	(1.891)	(1.698)
TA EU2 × Continental			− 2.846*
			(1.380)
TA EU2 × Nordic			− 14.81***
			(1.677)
TAvEU2 × Liberal			− 18.04***
			(3.532)
EU2 enlargement	3.130	4.928	0.493
	(4.476)	(5.267)	(4.348)
Native self-employment rate		− 0.361	0.230
		(1.345)	(0.551)
Share EU2		− 6.373	− 7.779***
		(4.147)	(2.054)
Unemployment		0.0571	− 0.0566
		(0.463)	(0.204)
GDP per capita PPA		0.000240	0.0008***
		(0.000254)	(0.000204)
Employment protection legislation		0.414	− 0.531
		(6.290)	(2.768)
Constant	11.72**	9.944	− 12.09
	(4.572)	(12.76)	(9.190)
Observations	238	234	234
*R*-squared	0.121	0.159	0.318
Number of countries	15	15	15

All models shown include country and year fixed effects. Robust standard errors, clustered at the country level, in parentheses. Significance at ***p < 0.01, **p < 0.05, *p < 0.1. Data for Finland and Sweden includes both EU2 and EU8, as available in the EULFS

However, an analysis of the overall sample might obscure variation between countries, since, as previously mentioned, there are significant differences in the socio-economic regime and institutional configuration of each country analysed and there was substantive variation in terms of the attractiveness of a particular country and the restrictiveness of the TAs it put in place. Thus, in order to disentangle the effect of the TAs on EU2 self-employment rates across different countries, and to make the most of the relatively small number of country-year observations in our sample, we consider the four typologies of the regimes of capitalism previously discussed, which we interact with our independent variable. The results, presented in model 3, point to a differentiated effect across the four typologies.^4^ Specifically, the removal of transitional arrangements does not seem to have affected the self-employment rates of EU2 nationals in the Southern European countries, whose effect is picked up by the general TAEU2 variable in Model 3, but it has strongly and negatively affected self-employment rates in the three other types of capitalist regimes. These results suggest that self-employment was not used instrumentally and strategically by EU2 nationals (and their employers) as an entry point to labour markets in Southern countries, but it was used in the Liberal, Nordic, and Continental regime countries.

The lack of effect for Southern countries does not come as a surprise, since there were numerous sectors and professions exempted from restrictions especially in Italy and Spain, meaning that EU2 nationals did not “need” to turn to self-employment in order to access these countries’ labour markets. Rather, the strongest decrease in self-employment rates after the removal of transitional arrangements seems to have taken place in the Liberal country group, representing Ireland and the UK. One explanation might be that having experienced a largely unexpected and substantial inflow of EU8 nationals post-2004, which fulfilled their labour needs but in some cases posed infrastructure (schools, housing, hospitals) related challenges (Fihel et al. 2015), the UK and Ireland decided to implement more stringent labour market restrictions for EU2 nationals. The unintended consequence of this development seems to have been an upsurge in bogus self-employment as a labour market access route, and a subsequent decline once restrictions were removed and access to paid employment was no longer hindered. In the Nordic countries, the similarly strong and negative effect un self-employment rates is largely driven by Denmark, the only country in the group to have implemented rather restrictive TAs, fearing an “unintended use of social security benefits” and “undue pressure on wages” (Wright 2010, 161). The still negative and significant but somewhat lower in magnitude effect in Continental countries might be explained by the co-existence of stringent and well enforced TAs with some exemptions in countries such as Germany, Austria and the Netherlands.

Lastly, the share of EU2 nationals in a country’s population seems to have a negative and significant effect on their self-employment rates in model 3, suggesting that the presence and resources of networks might have helped with finding paid employment. Contrariwise, the level of GDP per capita exhibits a small but positive effect on self-employment rates.

Table 4 presents the results of our analysis for the EU8 nationals. In this case, both models 1 and 2 indicate that the removal of transitional arrangements did not have a significant effect on the self-employment rates of EU nationals across all countries, although the sign of the coefficients also suggests a negative relationship. Rather, model 2 suggests that the socio-economic conditions as proxied by our native self-employment rate variable, and the presence of networks are far more important in explaining variation in self-employment rates. Model 3, which considers variation in socio-economic and institutional regimes across the four VoC types, indicates a negative relationship between the removal of TAs and self-employment rates in the Continental, but not in the Nordic countries (Southern countries represent the baseline, picked up by the standalone TAEU8 variable, and Liberal countries are not included because they did not implement TAs). These results suggest that EU8 nationals turned to self-employment to access the labour markets of Continental countries such as Austria, Germany, or the Netherlands, all of which were historically attractive destinations for this group, but did not do so in the Nordic countries of Finland and Denmark. This result might be explained by their lower attractiveness as destination countries, pointing to the fact that labour demand is also needed to attract migrants, and that geographical distance and language skills can act as barriers (Galgóczi et al. 2011).

**Table 4:** The effect of TA removal on EU8 self-employment rates

Variables	(1)	(2)	(3)
TAE U8	− 0.367	− 1.023	− 2.931
	(3.071)	(2.678)	(2.416)
TAE U8 × Continental			− 7.341*
			(4.009)
TAE U8 × Nordic			− 8.139
			(4.678)
Native self-employment rate		1.361*	1.257*
		(0.653)	(0.630)
Share EU8		− 3.518**	− 2.670
		(1.390)	(1.526)
Unemployment		0.386	0.299
		(0.257)	(0.230)
GDP per capita PPA		0.000177	0.000191
		(0.000173)	(0.000166)
Employment protection legislation		4.608	6.084
		(3.999)	(4.049)
Constant	9.894***	− 29.44*	− 31.82*
	(2.762)	(16.28)	(16.87)
Observations	237	233	233
*R*-squared	0.064	0.193	0.223
Number of countries	15	15	15

All models shown include country and year fixed effects. Robust standard errors, clustered at the country level, in parentheses. Significance at ***p < 0.01, **p < 0.05, *p < 0.1. Data for Finland and Sweden includes both EU2 and EU8, as available in the EULFS

## Conclusion

In this article, we have investigated whether and how the removal of transitional arrangements has affected the self-employment rates of EU2 and EU8 nationals in the EU15 member states. Our hypothesis has been that, if EU2 and EU8 nationals have turned to self-employment as a means to evade the transitional arrangements in place, leading to an upsurge in (bogus) self-employment rates, then the removal of transitional arrangements should lead to a corresponding decrease.

Our results strongly indicate that this has been the case for EU2 nationals, as we consistently find a negative relationship between the removal of TAs and their self-employment rates across the entire sample. The switch from self-employment to employment, as indicated by our results, implies that self-employment was a means for EU2 nationals and employers to comply with the existing rules and regulations, and not necessarily a reflection of the entrepreneurial spirit of this migrant group or the employment conditions in the receiving countries. Furthermore, and importantly, zooming in on countries grouped according to the VoC framework reveals a more nuanced picture, with considerable variation in terms of the magnitude of this negative effect across all four types of regime.

On the other hand, we do not find a significant effect of the removal of TAs on the self-employment rates of EU8 nationals, when we analyse the sample in its entirety. This result might reflect a “diversion” effect within the EU15 countries. That is, the transitional arrangements seemed to have diverted flows away from some traditional destinations of migration from Central-Eastern Europe, which have now applied restrictions, to countries which maintained their labour markets open (e.g. Ireland, UK) (Boeri and Brucker 2005; Barrell et al. 2010; Kahanec and Zimmermann 2010). Germany or Austria still experienced significant inflows from the EU8 nationals due to the relatively high demand for labour, historical ties, and network effects, but the transitional arrangements reduced their relative position among all the potential destination countries (Kahanec et al. 2016). To illustrate, while in Germany, the net gain of EU8 inflows between 2004 and 2006 was 2.5 times larger than in the 2000–2004 period (Brenke et al 2010), in the UK (which did not apply restrictions), the stock of EU8 immigrants increased from around 50,000 in 2003, to 704,000 in 2008, a 14-fold increase (United Kingdom Migration Advisory Committee 2008). Thus, the effect of the transitional arrangements on the self-employment rates of EU8 (and likely EU2) nationals seems to not only have depended critically on the specific measures that one member state has implemented, but also on the measures implemented by competing, alternative member state destinations. From a policy perspective, these developments highlight the need for policy makers to look beyond their own borders when implementing measures that aim to control and influence migration flows (Palmer and Pytliková 2015) and to anticipate how other countries’ policies will interact with their own and affect mobility and labour market decisions. Considering that networks play an important role in perpetuating migration patterns, with information and knowledge from fellow nationals weighing significantly in location decision-making, the distortion in the distribution of EU8 (and EU2) nationals across the EU15 member states resulting from the transitional arrangements is likely to have had long-term consequences (Fic et al. 2011).

Importantly, in the case of EU8 nationals too, zooming in on country groups reveals variation in terms of the impact of TA removal on self-employment rates across the different institutional regimes. This variation reflects the specific institutional configuration, socio-economic context, and labour demands of each country, which in turn are reflected in their degree of labour market openness post-enlargement and thus in the “need” for EU8 and EU2 nationals to use self-employment as a circumventing measure.

Our article makes several important contributions. Firstly, it adds knowledge to the existing literature on migrant self-employment, which has preponderantly focused on personal characteristics of migrants and available networks as determinants of self-employment, and less so on institutional and policy related factors. Secondly, it helps us better understand the role and effectiveness of labour market restrictions, revealing the importance of considering the effect they have in shaping the volume and skill composition of migrants, as well as their labour market trajectories and subsequent economic activities. Our findings suggest that restrictions do not necessarily stop immigration, but rather affect the channels people choose to enter, as Czaika and de Haas (2013) have previously asserted. Immigration is driven by strong social and economic forces that are bound to compete with migration regulations (Palmer and Pytliková 2015). Indeed, when there are strong pull and push factors in place — as were the significant wage gaps in this case — barriers seem to do little to stop immigration. Lastly, our findings suggest that the effectiveness of the transitional arrangements, which were put in place to manage the inflow of new EU nationals, might have been undermined by the exemption of self-employed individuals. Put differently, the effectiveness of restrictive measures as tools that enable EU countries to tightly regulate the labour market access and outcomes of nationals from new member states is bound to be limited as well as variable across countries and over time (Ruhs and Wadsworth 2018).

All of this knowledge is important in view of a possible imposition of similar transitional arrangements in case of future EU enlargements, the integration of other regional blocs such as ASEAN, USMCA, or MERCOSUR, or in case of restrictions on worker mobility during health crises, such as the COVID-19 pandemic. If the transitional arrangements were indeed circumvented by both individuals and employers through self-employment as our findings indicate, then we ought to know more about their implications for labour mobility in the EU, the ability of migrant workers to find employment in incumbent member states, the employers’ ability to fill vacancies with qualified individuals, and the socio-economic impacts of this (matching) process.

## Figures and Tables

**Fig. 1:**
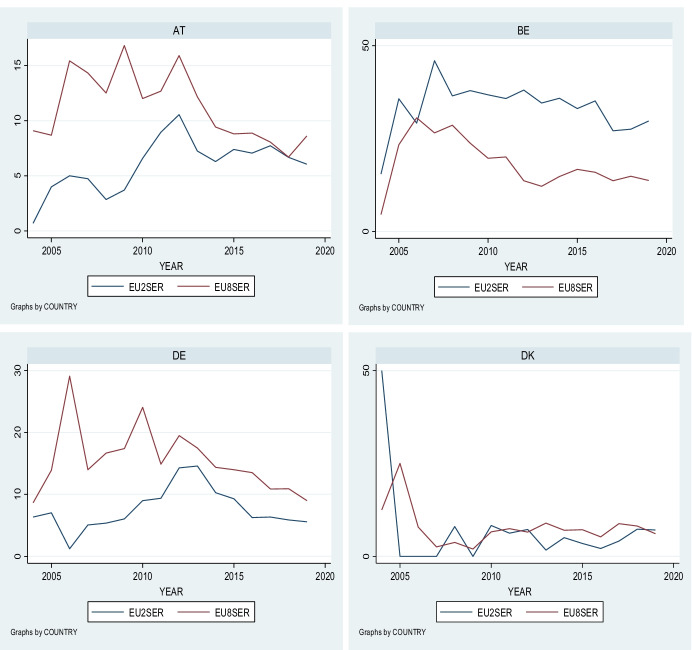

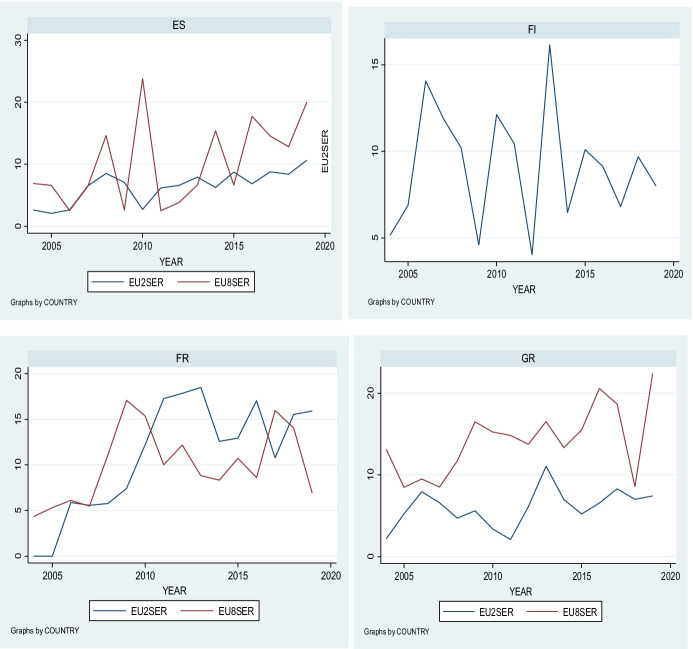

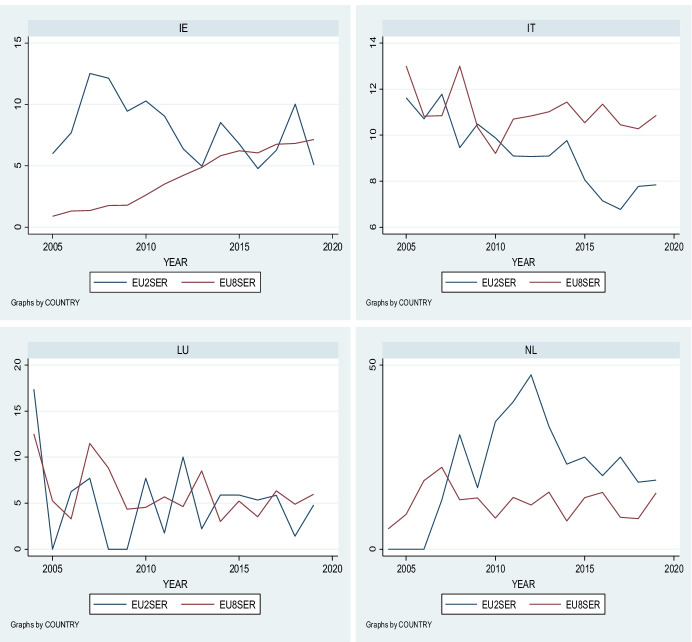

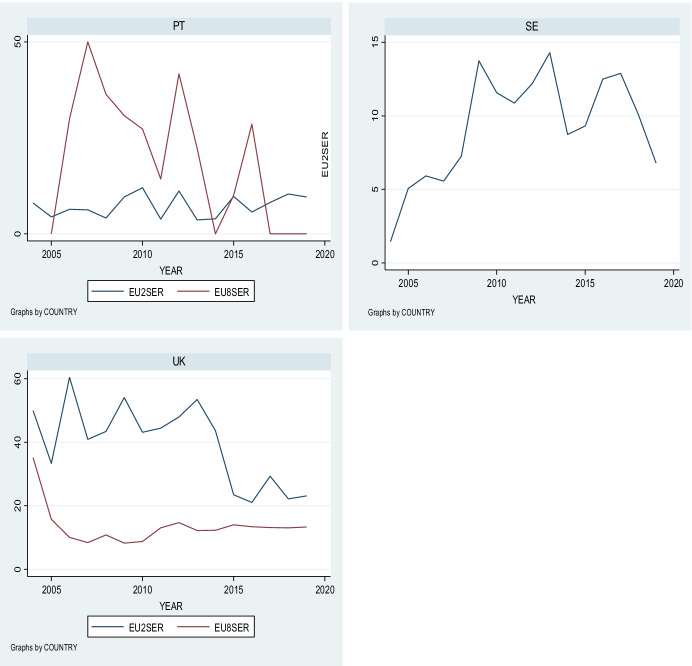
Self-employment rates of EU2 and EU8 nationals, by country, 2004–2019

**Fig. 2:**
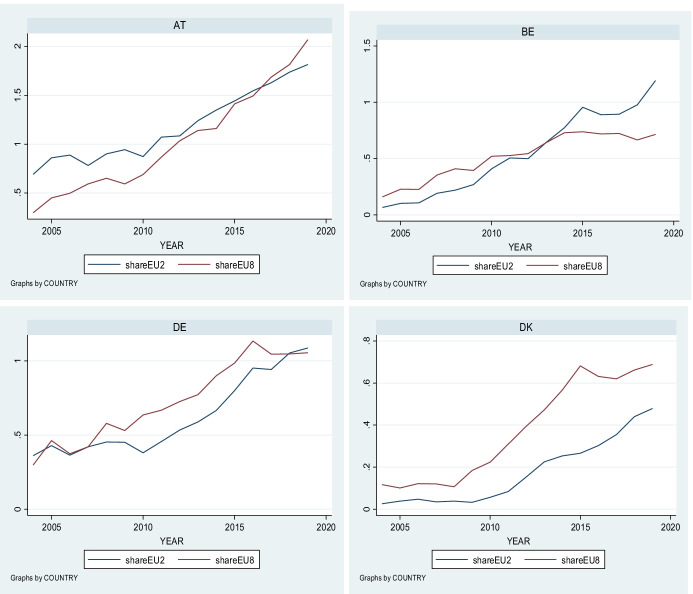

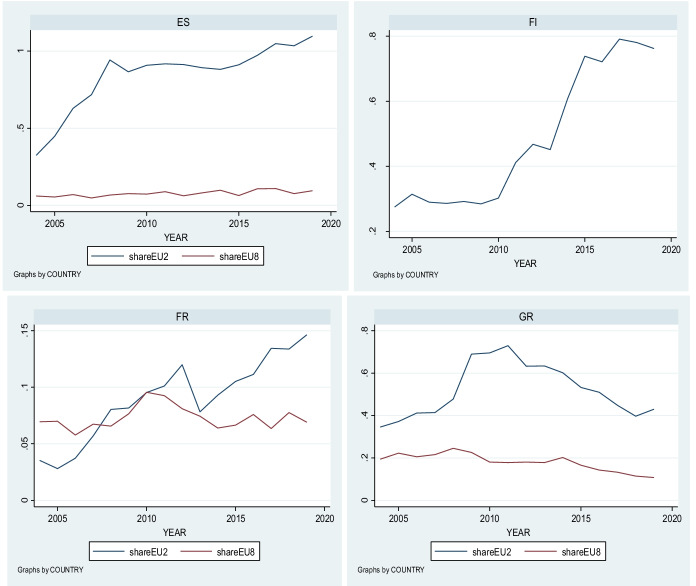

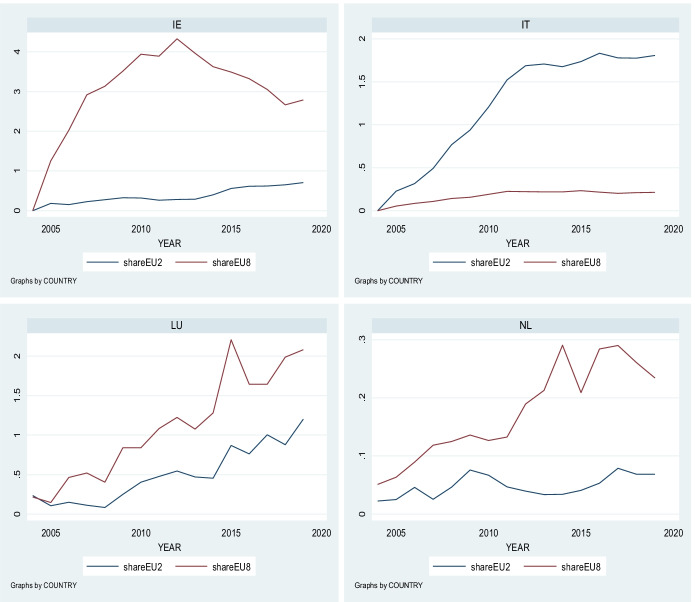

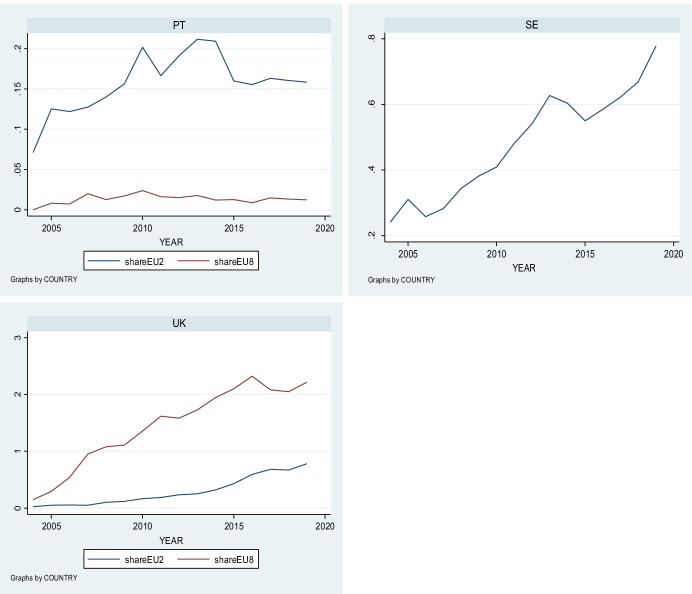
Share of EU2 and EU8 nationals in the total population, by country, 2004–2019

## Footnotes

1 The EU2 countries include Bulgaria and Romania. The EU8 countries include Czechia, Estonia, Hungary, Latvia, Lithuania, Poland, Slovakia, and Slovenia. Cyprus and Malta also joined the EU in 2004, but are omitted here because their citizens were not subjected to transitional arrangements. The EU15 countries include Austria, Belgium, Denmark, Finland, France, Germany, Greece, Ireland, Italy, Luxembourg, Netherlands, Portugal, Spain, Sweden, and the UK.

2 For more information, please see http://europa.eu/rapid/press-release_MEMO-11-773_en.htm.

3 The results of the analysis with or without Finland and Sweden do not differ significantly.

4 Southern countries represent the baseline.
